# A ResNet50-DPA model for tomato leaf disease identification

**DOI:** 10.3389/fpls.2023.1258658

**Published:** 2023-10-16

**Authors:** Jin Liang, Wenping Jiang

**Affiliations:** School of Electrical and Electronic Engineering, Shanghai Institute of Technology, Shanghai, China

**Keywords:** tomato leaf image, disease identification, deep learning, convolutional neural network, feature extraction

## Abstract

Tomato leaf disease identification is difficult owing to the variety of diseases and complex causes, for which the method based on the convolutional neural network is effective. While it is challenging to capture key features or tends to lose a large number of features when extracting image features by applying this method, resulting in low accuracy of disease identification. Therefore, the ResNet50-DPA model is proposed to identify tomato leaf diseases in the paper. Firstly, an improved ResNet50 is included in the model, which replaces the first layer of convolution in the basic ResNet50 model with the cascaded atrous convolution, facilitating to obtaining of leaf features with different scales. Secondly, in the model, a dual-path attention (DPA) mechanism is proposed to search for key features, where the stochastic pooling is employed to eliminate the influence of non-maximum values, and two convolutions with one dimension are introduced to replace the MLP layer for effectively reducing the damage to leaf information. In addition, to quickly and accurately identify the type of leaf disease, the DPA module is incorporated into the residual module of the improved ResNet50 to obtain an enhanced tomato leaf feature map, which helps to reduce economic losses. Finally, the visualization results of Grad-CAM are presented to show that the ResNet50-DPA model proposed can identify diseases more accurately and improve the interpretability of the model, meeting the need for precise identification of tomato leaf diseases.

## 1 Introduction

In 2021, China’s tomato production reached 67.63 million tons, accounting for 35% of the world’s total output, which is a very popular garden plant (Bhatkar et al., 2021; Jing et al., 2023; Liu et al., 2023). However, plant disease resistance needs to be faced by all crops in large-scale production, and various diseases can damage crops in any given growing season, resulting in reduced yield or lower quality. For example, early blight, a fungal disease that is a common type of disease in field-grown tomatoes, causes round or elongated brown lesions to form on infected plant tissue (Paul et al., 2019; Hu and Zhang, 2023). Late blight causes infected leaves to turn pale green to brown and eventually to wither and die (Arafa et al., 2022). Therefore, it is very important to quickly and accurately identify tomato leaf disease types and provide timely targeted treatment. The traditional research on disease recognition is to preprocess crop disease pictures first, and then use manual recognition methods to classify diseases. However, this method is highly dependent on professional knowledge and the cost of expert diagnosis is high (Manavalan, 2021; Li et al., 2022; Thakur et al., 2022). Meanwhile, it is time-consuming, labor-intensive and highly subjective, which is prone to misclassification. Therefore, a method that can accurately identify tomato leaf diseases is urgently needed.

With the rapid development of computer vision and artificial intelligence, the study on crop disease recognition based on image data has received extensive attention (Al-Wesabi et al., 2022), and the recognition accuracy has also been greatly improved. According to the extraction method of disease features in images, it can be divided into method based on machine learning and method based on deep learning (Saleem et al., 2019; Vishnoi et al., 2020). In terms of machine learning, Qin et al. (2017) used the K-median clustering algorithm and the segmentation method of linear discriminant analysis to obtain the image of the diseased area, and then extracted texture for detecting alfalfa leaf disease by SVM model. Finally, the identification accuracy rate is 80%. Sghair et al (Sghair and Naisbitt, 2017). segmented leaf disease areas by filtering and thresholding color features, and then detected rice leaf disease types, with an accuracy rate of 96.6%. However, the leaf texture and shape characteristics are not discussed, resulting in the loss of leaf information. Yao et al. (2009) used the GLCM algorithm to extract the characteristics of rice leaf area, perimeter, contrast, and leaf length and width. Then the SVM algorithm is employed for classification and recognition, with an accuracy rate of 97.2%. While, disease types with similar textures are prone to misclassification. Wang et al. (2012) studied the grape and wheat diseases identification, where the K-means clustering algorithm is applied to segment the disease area, and the backpropagation network are designed as the classifier. The results showed that the accuracy of grape and wheat disease recognition is 97.14% and 100%, respectively. However, the features extracted in (Wang et al., 2012) have been combined many times to achieve the best recognition effect. How to find a suitable feature combination set is still a great challenge. Compared with manual recognition, the recognition accuracy of the machine learning-based method has been significantly improved, and this method is more interpretable for the model. However, when the amount of data is large, the performance of machine learning-based method is not satisfactory. Meanwhile, manual design on features is inevitable, which requires the empirical knowledge of domain experts and relies heavily on feature engineering. the above problems can be effectively solved by the deep learning-based methods with higher computational performance, especially on large-scale datasets. This method uses an optimized loss function to learn rules, avoiding manual design of rules, which is highly portable, and can be compatible with multiple platforms. Therefore, many scholars apply it to the identification of leaf diseases, taking advantage of its real-time, automatic feature learning and high accuracy, in order to take control measures in time to protect the growth of crops. For example, Elhassouny et al (Elhassouny and Smarandache, 2019). applied the MobileNet neural network to identify tomato leaf diseases, and proposed an embedded intelligent application for the algorithm, which greatly reduced the required computing resources, and the accuracy rate reached 88.4%. However, the algorithm has a small amount of data and only uses more than 7,000 images for recognition, thus, how to avoid overfitting is a potential problem. Liu et al. (2020) first used the Inception structure to enhance feature extraction performance, and then introduced a dense connection strategy to enhance feature propagation, and obtained a new CNN model DICNN. Compared with the traditional neural network, the recognition accuracy of the DICNN model has increased by 2.97%, which provides a new idea for the identification of grape leaf diseases. Zhao et al. (2021) introduced the attention mechanism, SENet, into ResNet50 to identify tomato leaf disease. Then the average recognition accuracy rate reached 96.81%, which strengthened the ability to extract information. However, the SENet module only focused on the channel attention mechanism, which caused the loss of image texture information during the transfer process. Liu et al. (2022) applied the ROI feature extraction algorithm to the DenseNet classification model, which showed that the ROI algorithm can highlight the lesion area of rice leaves and improve the recognition accuracy. Finally, the accuracy rate of rice leaf disease recognition reached 96% in (Liu et al., 2022). Ahmad et al. (2021) used the gray-level co-occurrence matrix to extract the features of plant diseased leaves, and strengthened the extraction of image texture information. The research results showed that the classification accuracy rate reached 98.79%. Faisal et al. (2023) proposed a plant leaf disease classification algorithm DFNet that combines dual pre-training models (MobileNetV2, NASNetMobile), whose recognition accuracy for corn leaf disease and coffee leaf disease was 97.53% and 94.65%, respectively. Zhou et al. (2021) proposed the RRDN model for the disease identification of tomato leaves, which combined the deep residual network and the dense network to improve the calculation accuracy and reduce the number of parameters. The results show that the average identification accuracy rate reached 95%. Zhao et al. (2022) introduced the residual structure as a convolutional block into the Inception network, which alleviated the problem of gradient disappearance. Simultaneously (Zhao et al., 2022), embedded an attention mechanism in the model to enhance the feature extraction performance of plant leaves, and experimental results show that the algorithm has higher recognition accuracy and fewer model parameters.

To sum up, the convolutional neural network is conducive to the accurate identification of plant leaf diseases, liberates the labor force, and realizes the development of smart agriculture (Chin et al., 2023). Moreover, many scholars have improved the recognition accuracy by strengthening the model’s feature extraction performance. However, the effect still needs to be further improved. In this regard, this paper studies the tomato leaf disease recognition problem, where the novel ResNet50-DPA model is proposed, which effectively enhances the ability to extract image features and improves the recognition accuracy. The main contributions of the paper are summarized as follows:

(1) In ResNet50-DPA model, the improved ResNet50 is obtained by using cascaded atrous convolution instead of the first layer of convolutions in the ResNet50 residual network, for capturing tomato leaf characteristics with different scales.
(2) In this model, a novel DPA mechanism is proposed, which contains a channel attention module and a spatial attention module where the stochastic pooling is innovatively introduced. Meanwhile, two convolutions with one dimension are employed to replace the MLP layer in DPA. This mechanism helps to extract information of important areas in the image, and effectively reduces the damage to the leaf information.
(3) The enhanced tomato leaf feature map is obtained by inserting the above DPA into the residual module of improved ResNet50, which facilitates obtaining more comprehensive information, and then quickly and accurately identifying tomato leaf disease types and reducing crop losses.

The rest of this paper is as follows: Section 2 introduces the data set used and describes the proposed model in detail. In Section 3, grouping experiments are carried out for the proposed model, the experimental results are analyzed, and the model is visualized. Section 4 summarizes and explains the future work.

## 2 Methodology

### 2.1 Data preprocessing

**Figure 1:**
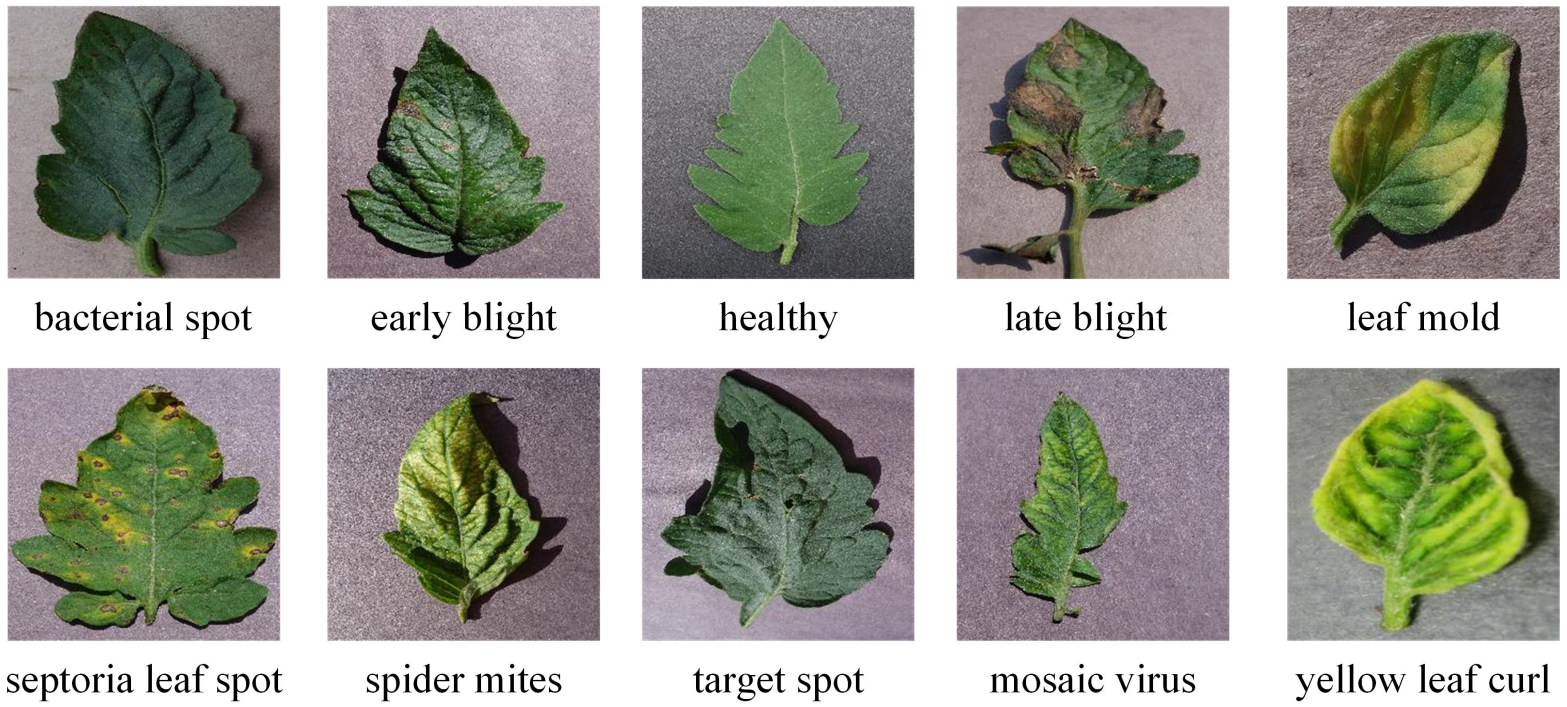
Sample image of tomato leaf diseases.

The quality of images has a significant impact on the image processing, feature extraction, and ultimately tomato leaf disease recognition. Plant Village, an open data set on the Internet, used in this paper, which a total of 14 plant varieties, each of which contained different leaf disease types. Among them, tomato leaf disease images accounted for 26% of the total data set, with a total of 10 categories, including 1 healthy category and 9 diseased category images, which was much larger than the data of other plant species. Therefore, tomato leaf disease images were selected as the research object in this paper. **Figure 1** shows all categories of tomato leaf disease sample images included in this dataset.

**Figure 2:**
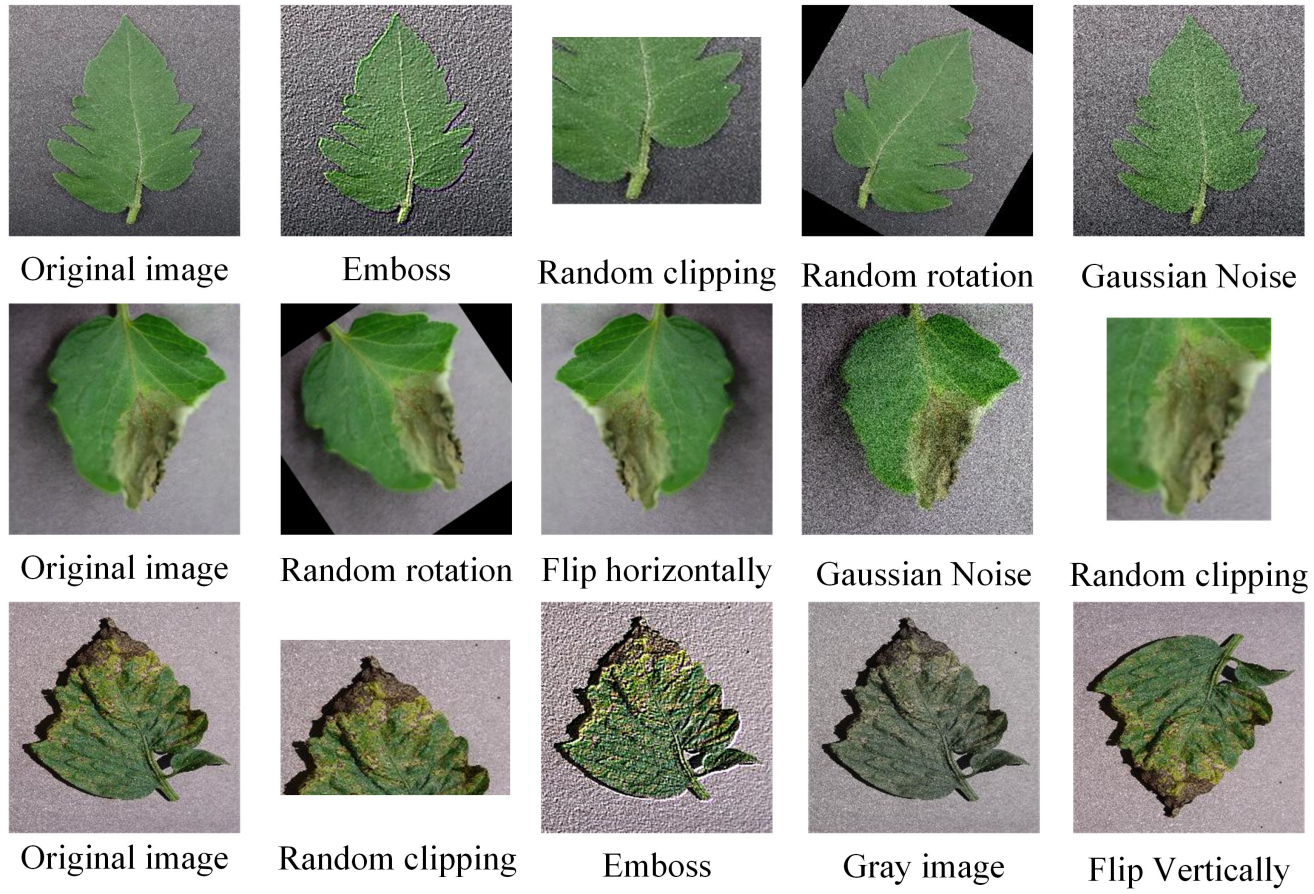
Sample image enhancement example.

In deep learning, increasing the amount of data used by the training neural network has an important impact on the final recognition effect. The model trained after enriching the data set has strong generalization ability and can ensure the diversity of the data to avoid overfitting during the training process. Therefore, this paper adopts imgaug image enhancement library to expand the data set. The emboss enhancement outlines the outline of the image, highlights the changing part of the image, and fade the same gray level part to reflect the depth. Image equalization in image enhancement is denoising by using the residual information prevalent in natural images. In this paper, the specific operation is to make the pixel values originally distributed in a concentrated way evenly distributed to all possible ranges, so that the contrast and brightness of the image can be improved and the details of the tomato leaf image can be better observed (Tang and Isa, 2017). In **Figure S1**, is the histogram before image equalization processing, and the height in the figure represents the total frequency of this pixel value. It can be seen that most of the pixel values before processing are concentrated in a region, and **Figure S1** is the histogram after image equalization processing, where the frequency of the pixel value 70 decreases significantly, while the frequency of the other pixel values increases, and is evenly distributed everywhere. **Figure 2** below is the effect of the randomly selected tomato leaf sample image enhanced by the above data.

After image enhancement and equalization processing, the total data reached 22,930 images. 20% of the total data set was divided into test sets, and the detailed values of the final data set were shown in **Table 1**. A total of 10 tomato leaf disease categories were included in the dataset, including 18,345 images in the training set and 4585 images in the test set.

**Table 1:** Detailed information on tomato leaf disease data set.

Tomato leaf category	Total	Train	Test
bacterial spots	2127	1702	425
early blight	2400	1920	480
healthy	2407	1926	481
late blight	2314	1851	463
leaf mold	2352	1882	470
septoria leaf spot	2181	1745	436
spider mites	2176	1741	435
target spot	2284	1827	457
mosaic virus	2238	1790	448
yellow leaf curl	2451	1961	490

### 2.2 ResNet50-DPA model

Problem statement:

For leaf disease recognition, most of the existing studies improve the recognition accuracy by deepening the number of network layers or adjusting parameters. However, by observing the data set, we found that there were only slight differences between different types of tomato leaf diseases, so strengthening the feature extraction capability is our focus to improve the recognition performance. To this end, firstly, On the one hand, the atrous cavity convolution is added to the input layer of the model to obtain information of different scales; Then, on the other hand, the dual-path attention mechanism is introduced into the residual module of the residual network to make the extracted information more comprehensive.

Model introduction:

The ResNet50-DPA model proposed in the paper consists of two parts: DPA and improved ResNet50. The dual-path attention mechanism is divided into two parts: channel attention module and spatial attention module, where the stochastic pooling is innovatively introduced. The addition of spatial attention module can effectively reduce the problem of image texture information loss caused by using only channel attention module. Different from the existing studies, the stochastic pool method is introduced in the two attention modules of channel and space, which can effectively eliminate the influence of non-maximum value in the feature graph. At the same time, the MLP layer is replaced by two one-dimensional convolution layers, which reduces the computation and avoids the destruction of tomato leaf information. In addition, based on the ResNet50, the model proposed in this paper innovatively replaces the first layer of 7×7 convolution in the original model with a cascade of atrous convolution, which can help us obtain tomato leaf features at different scales, facilitate the extraction of more comprehensive information, and make the final recognition more accurate. The following sections expand on DPA and the improved ResNet50.

#### 2.2.1 The DPA mechanism

Attention mechanism is a technique used to enhance the model’s attention to different parts of the input image, which improves the performance and generalization ability of the model by giving different weights to each area of the image. SENet, published in 2018, is a representative work that applies attention mechanism to channel dimension. Based on the SENet module, this paper proposes a novel attention mechanism, the DPA mechanism. Different from the SENet module, which only uses global average pooling, resulting in the loss of image texture information in the process of passing spatial information to the channel. On this basis, this paper introduces maximum pooling and stochastic pooling in DPA. The introduction of maximum pooling effectively reduces the problem of information loss (Chen et al., 2021), while stochastic pooling can eliminate the influence of non-maximum values and improve the model’s ability to perceive details, thus improving the model’s performance. Stochastic pooling uses the feature graph values in each channel divided by their sum to get a probability matrix. Random sampling and selection are performed according to the size of the probability values. The sampling results are positively correlated with the size of the values, and the probability of being selected is also greater if the element value is larger, instead of always taking the maximum value as in maximum pooling. The specific calculation process of stochastic pooling is as follows:

Firstly, the element values in each square in the pooled area are divided by the sum of the element values in all squares to obtain the probability values of each element. See Equation 1, where k represents the index of the element values.


(1) $$P=\left(p_{i}\right)=\frac{X_{i}}{\sum_{k\in R_{j}}X_{k}}$$

Then, position l is selected in the pooling region according to probability P, as shown in Equation 2.


(2) $$A_{j}=X_{l},l∼P\left(p_{1},\ldots ,p_{\left|R_{j}\right|}\right)$$

**Figure 3:**
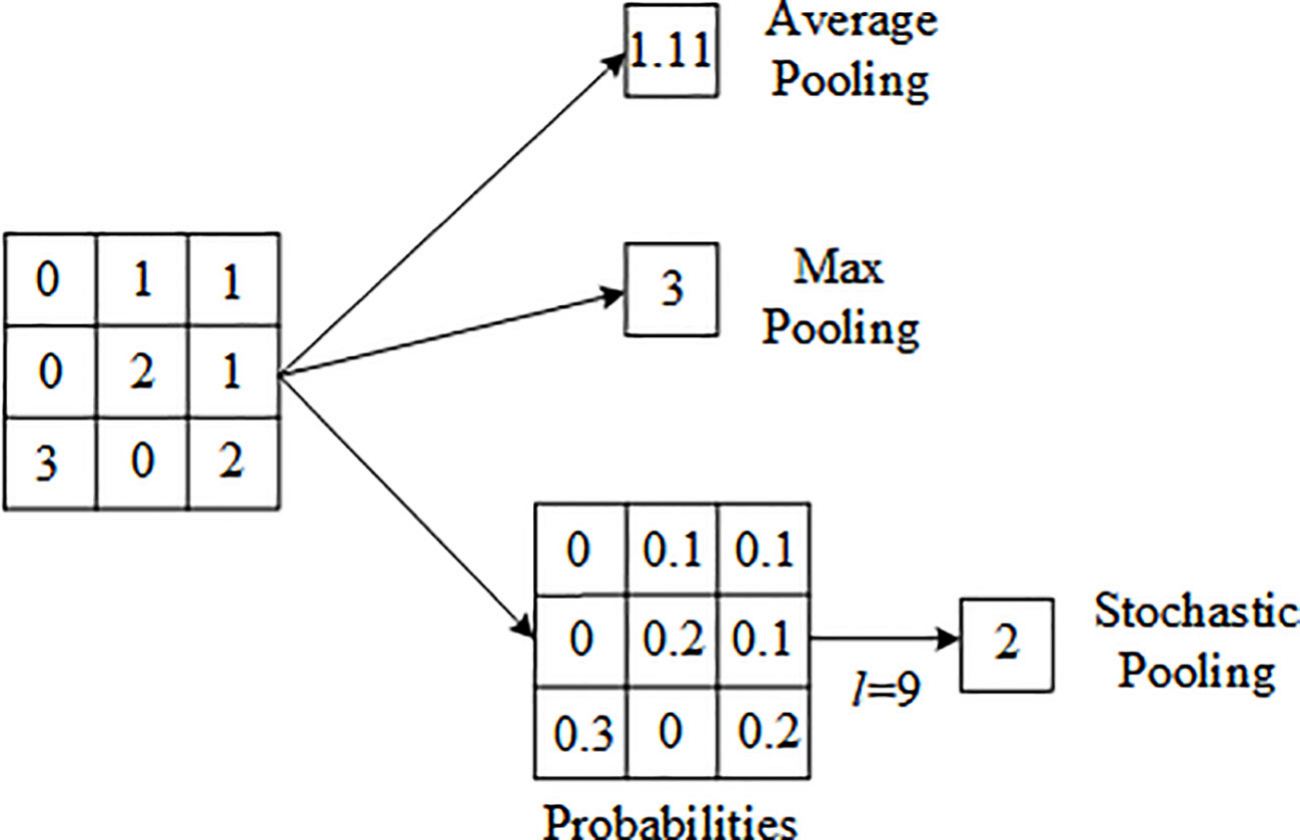
Examples of different pooling operations.

Finally, the final value is obtained according to the position comparison. **Figure 3** below shows the results obtained by using the three pooling operations respectively. It can be seen that the average and maximum pooling results are 1.11 and 3. The stochastic pooling first generates the probability graph, and then determines the position l. The final output result is 2, and the non-maximum value is selected.

**Figure 4:**
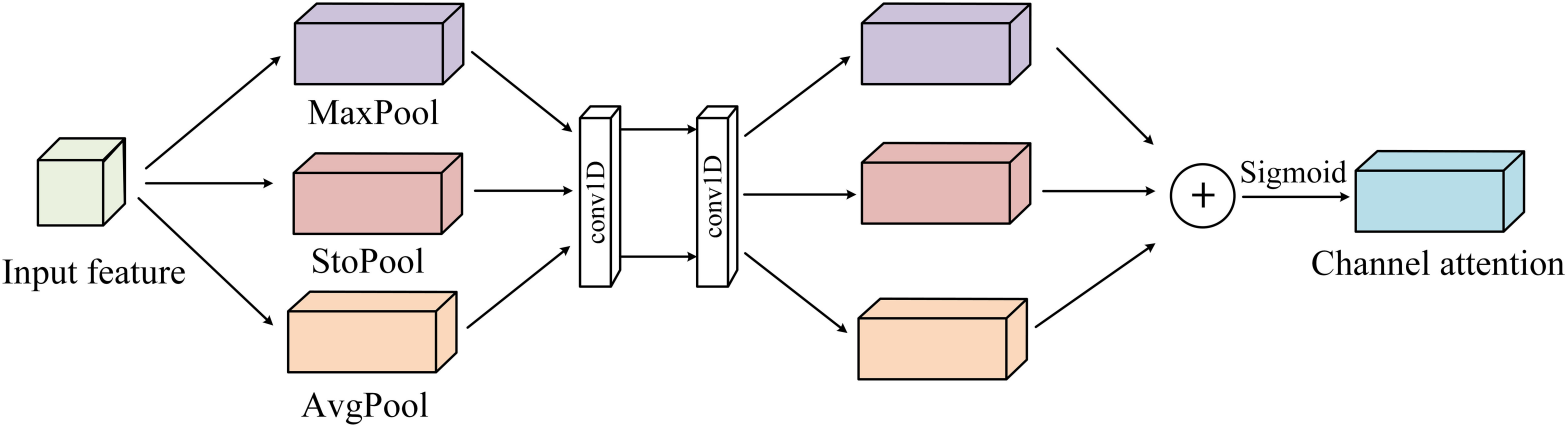
Channel attention module in DPA.


The structure diagram of the channel attention module in DPA is shown in **Figure 4** below. The addition of stochastic pool is conducive to eliminating non-maximum values, and at the same time, the model has stronger generalization ability and better regularization effect. Then, two one-dimensional convolution layers are used to replace the MLP layer. Since the one-dimensional convolution does not involve dimensionality reduction operation, damage to tomato leaf feature information can be avoided. Finally, the three spatial information descriptors generated are added and activated by the sigmoid to obtain the final channel attention module, as shown in Equation 3.

(3) $$M_{c}\left(F\right)=\sigma \left(conv\left(Max\left(F\right)\right)+conv\left(Sto\left(F\right)\right)+conv\left(Avg\left(F\right)\right)\right)$$

**Figure 5:**
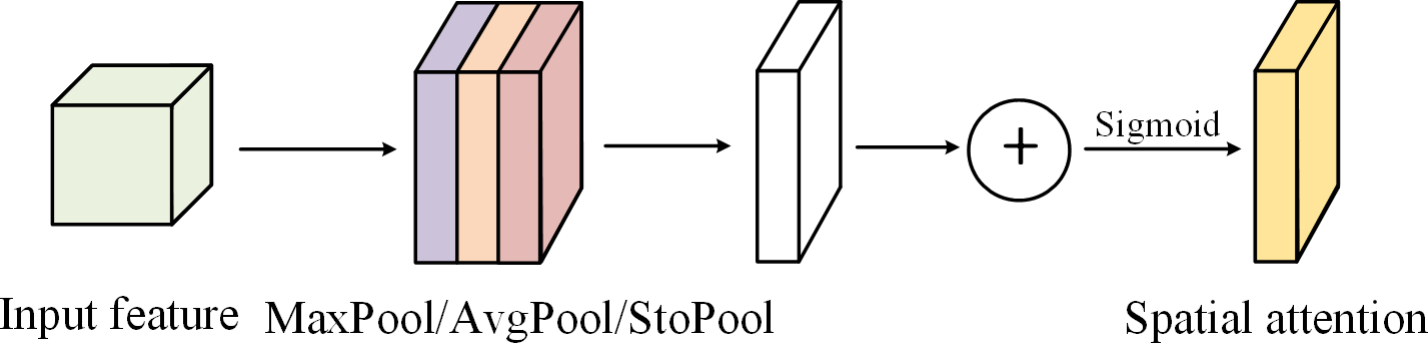
Spatial attention module in DPA.


The channel attention module in DPA mainly focuses on the relationship between channels of features, while the spatial attention module pays more attention to which part of the graph is more influential in the whole feature graph (Cao and Yuan, 2022). Similar to the channel attention mechanism, the spatial attention module of DPA also uses stochastic pooling operations, as shown in **Figure 5**. The main idea is to carry out global average pooling, global maximum pooling and stochastic pooling along channel dimensions, and then splicing the obtained results, using the 7×7 convolution layer to reduce dimension, and then obtaining the attention feature map of the spatial domain through sigmoid activation function. See the following Equation 4 for detailed description.

(4) $$M_{s}\left(F\right)=\sigma \left(f^{7\times 7}\left(\left[Avg\left(F\right);Max\left(F\right);Sto\left(F\right)\right]\right)\right)=\sigma \left(f^{7\times 7}\left(\left[F_{avg}^{s};F_{max}^{s};F_{sto}^{s}\right]\right)\right)$$

In the above two formulas, σ represents sigmoid operation. For the specific formula, see Equation 5. Avg represents global average pooling, Max represents global maximum pooling, Sto represents stochastic pooling, and f represents convolution operation.


(5) $$\sigma \left(x\right)=\frac{1}{1+e^{-x}}$$

#### 2.2.2 Improved ResNet50 residual network

With the continuous development and progress of deep learning theory and computer hardware, more and more applications have been made in various fields of our lives, such as face recognition, language recognition, automatic driving technology, etc. (Kute et al., 2019), but existing problems have also slowly emerged. In deep learning, the more convolutional layers, the better the classification effect will be. Therefore, in order to obtain deeper features, the number of convolutional layers is constantly deepening. At the beginning, LeNet network only has 7 convolutional layers, AlexNet has 8 layers, and the later VggNet network contains 19 layers. With the passage of time, the depth exploration of neural network is constantly carried out, but when it reaches a certain degree, the accuracy rate decreases, and the recognition accuracy of neural network is gradually saturated, resulting in the problem of gradient disappearance. Subsequently, the residual network ResNet was proposed by He et al. By constantly stacking residual structures, the problem of gradient disappearance during model training was effectively alleviated (He et al., 2016). In this paper, ResNet50 was selected as the basic model in consideration of the experimental effect and calculation amount, and its network structure was shown in **Table S1**.

The structure diagram of the residual block is shown in **Figure S2**, where X represents the input feature matrix. After two 3×3 convolution operations, the output is F(x), which is called the residual function, and the final output F(x)+X is passed into the next residual module. However, in ResNet50, the residual function F(x) is changed to three-layer convolution, as shown in (B) in **Figure S2**. A 3×3 convolution is retained, and a 1×1 convolution layer is added before and after the convolution layer to achieve the functions of reduction and dimension increase so that the expression features of the network are better and the corresponding detection or classification performance is stronger when the number of layers is increased. In addition, 1×1 convolution is used in the residual network, which reduces the number of parameters in the network and also reduces the amount of computation to some extent.

**Figure 6:**
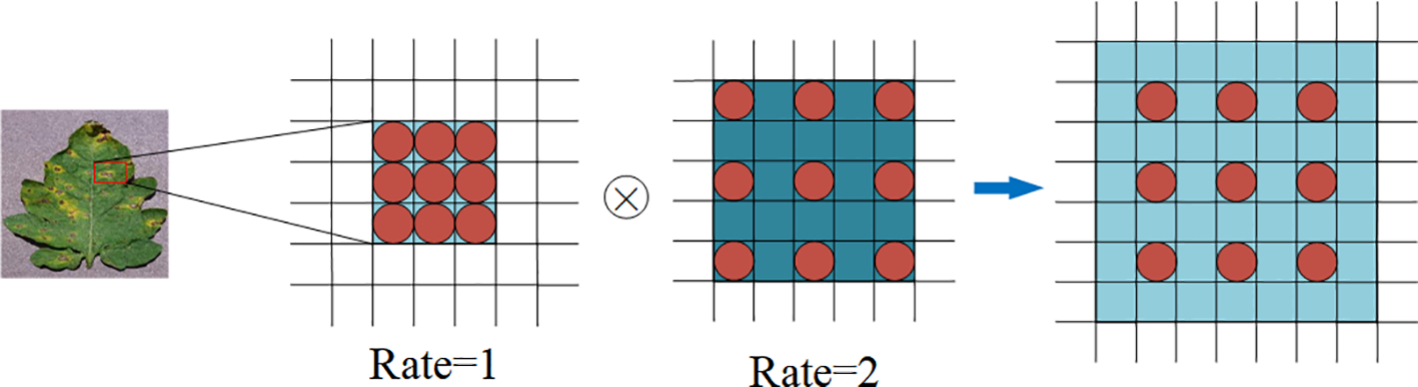
Extraction process of atrous convolution feature with different expansion rates.

In order to avoid the loss of spatial information when extracting feature graphs in the residual network model, this paper chooses atrous convolution replace the first layer 7×7 convolution in the ResNet50 network, which can enlarge the receptive field without increasing the number of parameters. **Figure 6** shows a schematic diagram of the atrous convolution with different expansion rates. In figure, the atrous convolution with the expansion rate of 1, 3×3 convolution kernel size has the same effect as the ordinary 3×3 convolution. For the atrous convolution with an expansion rate of 2, although the size of the convolution kernel does not change, the receptive field of the convolution has increased to 5×5, as shown in the dark blue part in **Figure 6**. The effect of ordinary 7×7 convolution nuclei can be achieved by concatenating these two 3×3 convolution nuclei with different expansion rates, as shown in the light blue part in **Figure 6**. Moreover, due to the different expansion rates of the two convolution nuclei, the sensitivity fields are also different, that is, multi-scale information is obtained, which helps to observe tomato leaf characteristics at different scales and extract more comprehensive information. The identification of subsequent tomato leaf diseases is also more accurate.

**Figure 7:**
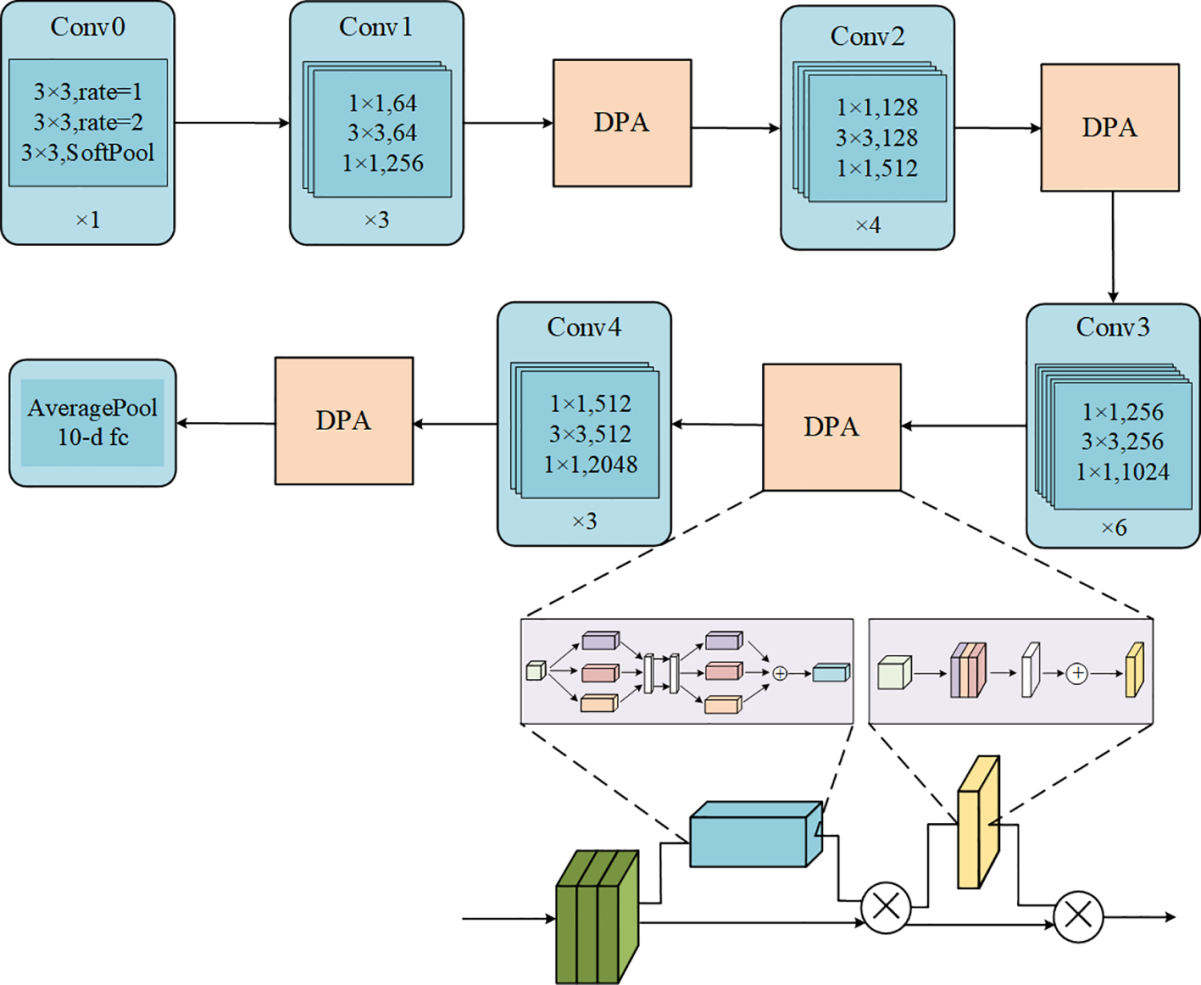
ResNet50-DPA network structure.

Finally, this paper adds the aforementioned DPA mechanism to the improved ResNet50 residual block structure, which can enlarge the weight of effective channels in the element layer, reduce the influence of redundant features with the help of DPA mechanism. It is worth mentioning that the 7×7 convolution in the first layer is replaced with a cascaded atrous convolution, which can extract tomato leaf features at different scales. Meanwhile, in this paper, the maximum pooling layer is removed and the soft pooling is chosen, which helps to retain more details in the original image. The final ResNet50-DPA structure diagram is shown in **Figure 7**.

### 2.3 Overall design for tomato leaf disease image recognition

**Figure 8:**
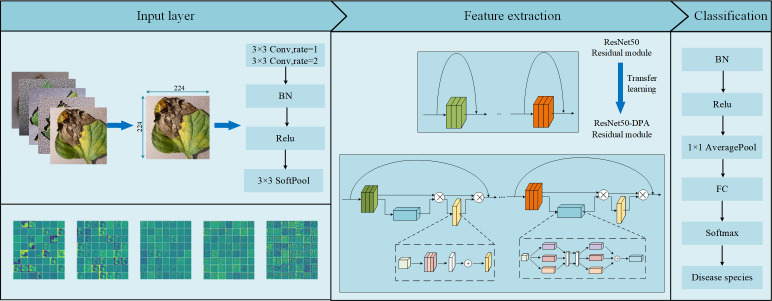
General design drawing.

**Figure 8** below is the overall design diagram of this paper. At the input layer of the model, the imgaug image enhancement library is first used to expand the data set. Due to the problem of random clipping, the data set at this time is RGB three-channel tomato leaf disease images with different sizes, and their sizes were uniformly adjusted to 224×224 as the model input. After atrous convolution and soft pooling operations, the DPA module was introduced into the residual module of the residual network to obtain the enhanced tomato leaf feature map. At last, after the normalization layer, the 1×1 global average pooling layer and the fully connected layer, the probability values of tomato leaves corresponding to different disease categories were calculated by the Softmax function, and the category of the maximum value is output as the classification result. Then, the classification and recognition of the final tomato leaf disease image is complete.

## 3 Experiment and data analysis

### 3.1 Experimental platform

The experiment is based on Windows 11 operating system, and the simulation software uses jupyter notebook compiler based on Python3.8 programming under Anaconda. The deep learning framework adopts Tensorflow architecture, which can support the model deployment on multiple GPUs, and the processor uses an i7 processor. RTX3050 graphics card set up the model proposed in this paper.

### 3.2 Evaluation criteria

In this paper, the accuracy rate, precision rate, recall rate and F1 score are as indicators to evaluate the performance of the proposed ResNet50-DPA model. In the following expressions, true positive (TP) represents the number of correctly classified positive samples, false positive (FP) represents the number of incorrectly classified negative samples, true negative (TN) represents the number of correctly classified negative samples, and false negative (FN) represents the number of incorrectly classified positive samples (Wang et al., 2017).

Accuracy: Accuracy is defined as the proportion of the number of correctly classified samples to the total number of samples, as shown in Equation 6.


(6) $$Accuracy=\frac{TP+TN}{TP+TN+FP+FN}\times 100\%$$

Precision: Precision is defined as the proportion of the number of positive samples with correct classification to the number of positive samples determined by the classifier, as shown in Equation 7.


(7) $$Precision=\frac{TP}{TP+FP}$$

Recall: Recall rate is defined as the proportion of the number of correctly classified positive samples to the number of real positive samples, as shown in Equation 8.


(8) $$Recall=\frac{TP}{TP+FN}$$

F1 value: refers to the harmonic average of precision rate and recall rate, see Equation 9.


(9) $$F1=2\times \frac{\left(Precision\times Recall\right)}{\left(Precision+Recall\right)}$$

### 3.3 Comparative analysis of hyperparameters

Hyperparameters are variables that define the network structure. In order to further improve the classification performance of the proposed model, it is necessary to discuss the values of some necessary parameters, such as batch size, loss function, and optimizer selection. In this section, we test within 50 epochs and employ the early stop technique. When the accuracy did not continuously improve within 5 epochs, the experiment was stopped to observe the effect of different parameter values on the classification effect. Meanwhile, three experiments are conducted to take the mean value of each parameter as the result. The values of the final major parameters are shown in **Table 2**. The following is a discussion and analysis of the specific parameters.

**Table 2:** Parameter value.

Parameter	Value
Batch size	32
Image size	224×224
Loss function	Cross-entropy
Optimization algorithm	Adam
Learning rate	0.001
Number of epochs	200

#### 3.3.1 Batch size

**Figure 9:**
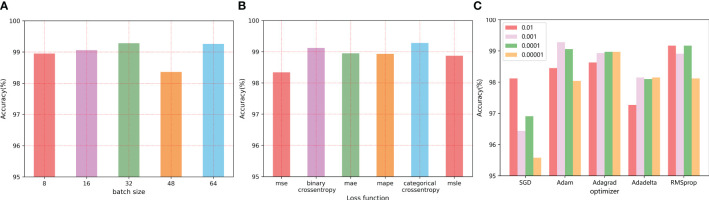
The influence of different parameter values on the final recognition effect: **(A)** The batch size **(B)** Loss function value **(C)** Optimizer selection.

Training batch size refers to the number of samples taken from the training set during each iteration training. The larger the value, the faster the model is trained, but too large a value makes it take up more memory. However, too small batch will lengthen the training time, and seriously may cause gradient shock, making the model convergence slow or unable to converge. Multiple literatures related to image classification are reviewed, and experimental results are observed with the common batch size. The accuracy of leaf disease recognition obtained with different values of batch size was shown in **Figure 9A**. With the increase of Batch size, the classification accuracy increased slowly, but the accuracy decreases as the value continued to increase. The classification effect is best when the value is 32.

#### 3.3.2 Loss function

The loss function is a function used to measure the gap between the real value and the predicted value, which is used to guide the model optimization direction and improve the prediction accuracy. When different loss functions are used, the model performance is shown in **Figure 9B**. In this paper, the cross-entropy loss function is finally selected, and compared with other loss functions, the cross-entropy loss function has better performance.


(10) $$L=\frac{1}{N}\sum_{i}L_{i}=\frac{1}{N}\sum_{i}-\left[y_{i}log\left(p_{i}\right)+\left(1-y_{i}\right)log\left(1-p_{i}\right)\right]$$

#### 3.3.3 Learning rate and optimizer

The learning rate can be used to control the updating speed of the weight of the neural network, usually with the order of 10, to search for the best learning rate on a logarithmic scale. However, it is impractical to continuously adjust the learning rate manually, so this paper uses typical learning rate values to test the classification performance, that is, from $10^{-2}$ to $10^{-5}$ (0.01, 0.001, 0.0001, 0.00001), At the same time with a number of different optimizer (SGD/Adam/Adagrad/Adadelta/RMSprop) are combined, and different combination of tomato leaf disease recognition results are shown in **Table S1** and **Figure 9C**, and finally set the model the optimizer to high calculation efficiency, low memory requirements of Adam optimizer, vector for 0.001.

### 3.4 Model performance comparison

In order to verify the recognition performance of the ResNet50-DPA model for tomato leaf diseases proposed in this paper, three comparison experiments are presented as follows.

**Figure 10:**
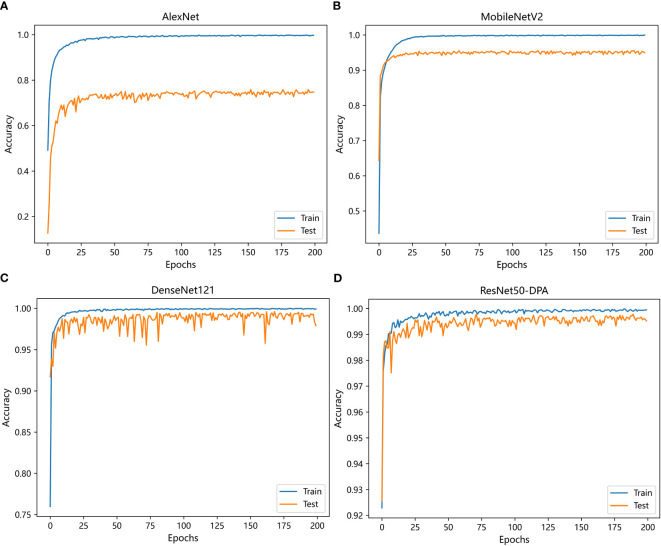
Variation curve of accuracy: **(A)** AlexNet **(B)** MobileNetV2 **(C)** DenseNet121 **(D)** ResNet50-DPA.

Comparison experiment 1:

The first set of experiments is trained with different deep learning models under the same data set and experimental environment. The compared models include AlexNet, MobileNetV2 and DenseNet121. The change curve of accuracy of each model in the training process is shown in **Figure 10**.

It can be seen from the curve trend in the **Figure 10** that with the increase of iterations, the prediction accuracy of each model has improved, among which AlexNet model has the slowest convergence speed and ResNet50-DPA model has the fastest convergence speed. Detailed experimental results are shown in **Table 3**. By observing the data in the table, we can see that the recognition accuracy of AlexNet, MobileNetV2 and DenseNet121 network models are 74.15%, 95.00% and 97.97%, respectively, while the recognition accuracy of the model proposed in this paper is 99.28%, which is significantly better than the previous three models.

**Table 3:** Comparison of disease recognition performance of different network models.

Model	Accuracy/%	Precision/%	Recall/%	F1/%
AlexNet	74.15	80.19	74.15	74.54
MobileNetV2	95.00	95.07	95.01	95.02
DenseNet121	97.97	98.03	97.97	97.97
ResNet50-DPA	99.28	99.29	99.28	99.28

**Figure 11:**
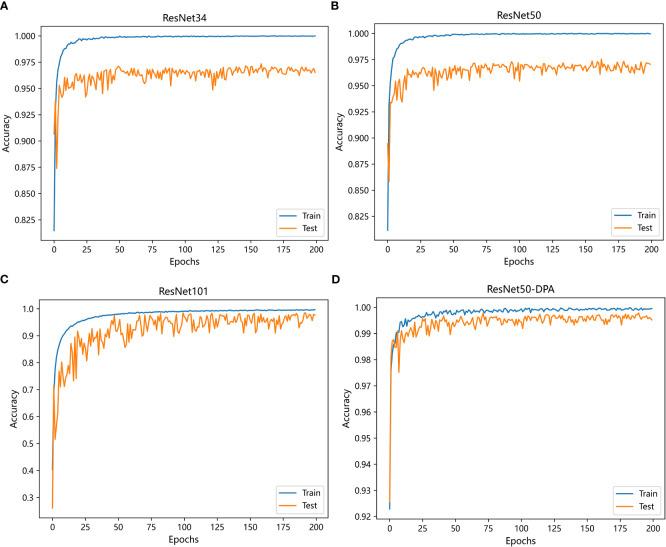
Change curve of ResNet series residual network accuracy: **(A)** ResNet34 **(B)** ResNet50 **(C)** ResNet101 **(D)** ResNet50-DPA.

Comparison experiment 2:

The second group of comparison experiments takes ResNet series residual network as the benchmark to observe whether the improved model has improved. **Figure 11** below shows the change curve of accuracy of the ResNet series residual network, and it can be seen that the proposed model still reaches convergence fastest. In **Table 4**, the recognition accuracy of the ResNet series residual network is 96.97%, 97.43% and 97.60% respectively, and ResNet101 has the best recognition effect, while the accuracy of ResNet50-DPA model reaches 99.28%, its classification performance is the best among all models, and its accuracy is 99.29%. The value of recall rate and F1 are both 99.28%, which is also better than other models, indicating that the DPA enhances the model’s ability to obtain information and can dig deeper features.

**Table 4:** Comparison of disease recognition performance of ResNet series residual networks.

Model	Accuracy/%	Precision/%	Recall/%	F1/%
ResNet34	96.97	96.98	96.97	96.96
ResNet50	97.43	97.43	97.43	97.42
ResNet101	97.60	97.69	97.60	97.60
ResNet50-DPA	99.28	99.29	99.28	99.28

Comparison experiment 3:

The third group of comparison experiments is compared with tomato leaf disease recognition algorithms proposed by different scholars in recent years, and the results are shown in **Table 5**. According to the **Table 5**, when the amount of data is relatively small, the classification algorithm proposed by Trivedi et al. has an accuracy of 98.49%. However, how to avoid overfitting is a potential problem. When the amount of data is similar, the model proposed in this paper has a higher accuracy due to obtaining multi-scale information by using cascades of atrous convolution when inputting images. Meanwhile, stochastic pooling is introduced into the dual path attention mechanism and one-dimensional convolution is used instead of MLP layer to reduce information loss, which is conducive to achieving better recognition accuracy.

**Table 5:** Compared with the existing methods of tomato leaf disease identification.

References	Year	Dataset	Images	Methods	Accuracy(%)
Zhao et al. (Vishnoi et al., 2020)	2021	Tomato leaf disease	18160	ResNet50+SeNet	96.81
Zhou et al. (Yao et al., 2009)	2021	Tomato leaf disease	13185	RRDN	95
Zhao et al. (Wang et al., 2012)	2022	Tomato leaf disease	18160	Inception + CBAM	95.2
Moussafir et al. (2022)	2022	Tomato leaf disease	14526	ResNet50 + EfficientNet	98.01
Anandhakrishnan et al. (Anandhakrishnan and Jaisakthi, 2022)	2022	Tomato leaf disease	18160	CNN	98.40
Theodora et al. (2023)	2023	Tomato leaf disease	18160	VGGNet	99.23
Rangarajan et al. (2018)	2018	Tomato leaf disease	13262	VGG16	97.29
Anandaet et al. (Ananda and Vandana, 2022)	2020	Tomato leaf disease	14421	VGG16	95.71
Ujawe et al. (Ujawe and Nirkhi, 2023)	2023	Tomato leaf disease	13262	AlexNet	97.49
Proposed	2023	Tomato leaf disease	22930	ResNet50 + DPA	99.28

**Figure 12:**
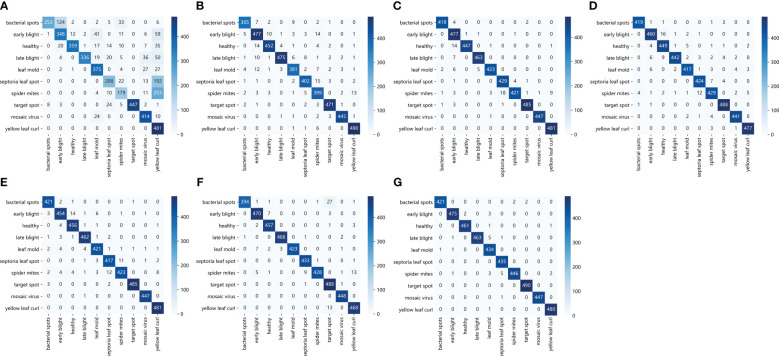
Confusion matrix: **(A)** AlexNet **(B)** MobileNetV2 **(C)** DenseNet121 **(D)** ResNet34 **(E)** ResNet50 **(F)** ResNet101 **(G)** ResNet50-DPA.

Except for the accuracy, this paper also verifies the ability of the proposed model to identify tomato leaf diseases from different perspectives. The classification results observed using the confusion matrix are shown in **Figure 12**. The horizontal and vertical coordinates in the figure represent the 10 types of tomato leaf diseases in the data set, and the elements on the main diagonal represent the number of correctly classified tomato leaf disease samples. It can be seen that the ResNet50-DPA model has only a few sample graph classification errors, which can achieve a good recognition effect for different disease types, while the number of classification errors of other models is much larger than that of the model proposed in this paper.

In addition, the detailed values of the classification results of the proposed model for different types of tomato Leaf diseases are presented in **Table 6**. It can be seen from the data in the table that the identification accuracy of the model for Leaf mold and Mosaic virus leaf diseases reaches 100%, while the accuracy of Leaf spot is only 97.09%. The model wrongly identified it as the type of spider mites, and the identification accuracy of other disease types is above 99%. The results shows that the research method proposed in this paper could achieves better recognition effect for different disease types, and could complete the accurate identification of tomato leaf diseases.

**Table 6:** Identification effect of the model for different disease types.

Disease type	Accuracy/%	Precision/%	F1/%	support
Bacterial spot	99.76	99.06	99.41	425
Early blight	99.37	98.96	99.16	480
Late blight	99.57	99.57	99.57	463
Leaf mold	100.00	98.51	99.25	470
Leaf spot	97.09	99.54	98.30	436
spider mites	98.42	1.00	99.20	435
Target spot	99.55	97.59	98.56	457
Yellow leaf curl	99.59	1.00	99.80	490
Mosaic virus	100.00	99.78	99.89	448
healthy	99.38	99.79	99.59	481
Macro avg	99.27	99.28	99.27	4585
Weighted avg	99.29	99.28	99.28	4585

### 3.5 Ablation experiments

In order to further verify the role of key modules in the model proposed in this paper, a set of ablation experiments are set up. The experimental results are shown in **Table 7**. It can be seen that the accuracy rate of the benchmark model ResNet50 is 97.43%, and the accuracy rate is increased by 0.42% after adding atrous convolution. When the channel attention mechanism is added to the residual module, the recognition accuracy rate is 98.21%; when the spatial attention mechanism is continued, the accuracy rate is 98.42%; when the DPA mechanism proposed in this paper is added, the accuracy rate can reach 99.28%, an increase of 1.85%, which is significantly improved compared with the basic model. These results show that the DPA module plays a crucial role in the overall framework, while the introduction of atrous convolution plays a role in the overall framework, but with limited effect. At the same time, the complexity of the model is measured by the number of parameters. It can be seen that the benchmark model ResNet50 has the smallest number of parameters. The model proposed in this paper increases the number of layers of the network by adding dual path attention mechanism and atrous convolution to the benchmark model ResNet50, resulting in an increase in the number of model parameters. However, from the final recognition accuracy of leaf disease, the effect of this model is ideal.

**Table 7:** Results of ablation experiments.

Description	Accuracy/%	Precision/%	Recall/%	F1/%	Params/M
ResNet50(baseline)	97.43	97.43	97.43	97.42	25.56
ResNet50 + atrous	97.85	97.48	97.79	97.63	25.58
ResNet50 + SENet	98.21	98.23	98.43	98.33	28.19
ResNet50 + CBAM	98.42	98.38	98.43	98.41	28.36
ResNet50 + DPA	99.28	99.29	99.28	99.28	30.78

### 3.6 Visualization of experimental results

In this paper, the output feature maps of different convolutional layers in the ResNet50-DPA model are visualized, as shown in **Figure S3**. It can be seen that with the increase in the number of convolutional layers, the extracted information became more and more abstract, macro information replaced the detailed information of the image, and the model learned more abundant features.

Finally, in order to see more directly which region the model focused on during the training process, gradient class activation mapping (Grad-CAM) is used to draw the heat map of the input tomato leaf disease image in this paper. The idea of Grad-CAM algorithm is to use a specific convolutional neural network architecture to generate a visual heat map (Selvaraju et al., 2017). The key is to obtain the weight by obtaining the partial derivative of the category confidence of the network output to the feature graph. The formula is shown in the following Equation 11, where c is an image category, $y^{c}$ is the gradient corresponding to the category, $A^{k}$ is the feature activation value, and the weight $\alpha_{k}^{c}$ of neurons can be obtained by calculation. The final heat map is obtained by weighted summation of the weight values of the categories corresponding to all feature graphs. See Equation 12 for the specific formula.


(11) $$\alpha_{k}^{c}=\frac{1}{Z}\sum_{i}\sum_{j}\frac{\partial y^{c}}{\partial A_{ij}^{k}}$$

(12) $$L_{Grad-CAM}^{c}=ReLU\left(\sum_{k}\alpha_{k}^{c}A^{k}\right)$$

**Figure 13:**
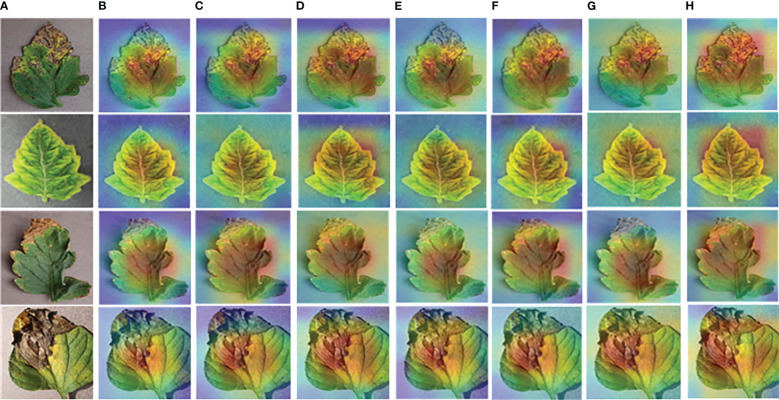
Visual heat map: **(A)** Original Image **(B)** AlexNet **(C)** MobileNetV2 **(D)** DenseNet121 **(E)** ResNet34 **(F)** ResNet50 **(G)** ResNet101 **(H)** ResNet50-DPA.

The heap map results obtained after Grad-CAM processing are shown in **Figure 13**. Some tomato leaf disease images are randomly selected, as shown in **Figure 13A**. The visualization results obtained by using different models are shown in **Figures 13B–H**. The visualization features obtained by ResNet50-DPA model are shown in **Figure 13H**. It can be seen that the model proposed in this paper pays more accurate attention to the disease region, and the Grad-CAM explains the ability of the model to identify leaf disease, which shows the region of interest of the model to the input image.

## 4 Discuss

In the identification of tomato leaf disease, a novel detection method based on ResNet50-DPA was proposed in this paper. How the proposed model accurately identifies disease types can be explained from the following two aspects.

On the one hand, the analysis is carried out according to the proposed detection method. The data enhancement technique is used to expand the data set to ensure the diversity of the data set and prevent the overfitting phenomenon. By replacing the first layer of convolution with a cascade of atrous convolution, atrous convolution can expand the range of receptive field and facilitate the extraction of features at different scales, instead of simply extracting blade features at the same scale. DPA is inserted into the residual module to enhance the extraction of important features. Three pooling operations are used in DPA, which not only reduces the problem of image information loss, but also eliminates the influence of non-maximum values in the model. The replacement of two one-dimensional convolution MLP layers also reduces the loss of information because it does not involve dimensionality reduction operations.

On the other hand, the analysis is based on the obtained results. Firstly, three sets of comparison experiments are carried out, and the experimental results show that the proposed model converges faster and has higher accuracy. Secondly, the confusion matrix are used to observe the classification performance of different disease categories. It can be seen that ResNet50-DPA has a good recognition effect on 10 tomato leaf disease types, and only a few samples are classified wrong. Finally, the Grad-CAM was used for visualization analysis. The Grad-CAM could highlight the most concerned regions of the model in the feature extraction process. The results showed that the proposed model could better focus on the disease regions of tomato leaves compared with other models, which verified the effectiveness of the proposed model.

In summary, although the current application scenarios are relatively limited, the models proposed in this paper can effectively identify different leaf disease types in limited scenarios, and the interpretability of the models is improved through visualization. The method proposed in this paper can help us to manage early disease, maintain crop growth, take control measures in time and reduce economic losses.

## 5 Conclusion

In this paper, a novel ResNet50-DPA model is proposed for tomato leaf disease recognition, which solves the problem of image information loss when extracting image features from conventional convolutional neural networks. In this method, the proposed DPA mechanism is introduced into the residual module of the residual network ResNet50 to enhance the ability of the network to extract details of tomato leaves. Then, cascades of atrous convolution are used to replace the first layer of convolution in ResNet50 to obtain leaf features at different scales and further improve the performance of the model. Finally, compared with the traditional methods, the experimental results show that the proposed model is superior to the common deep learning model, reaching an accuracy of 99.28%, which verifies the effectiveness of the proposed model for tomato leaf disease classification. In addition, the generalization ability and computing speed of the model proposed in the paper need to be further improved, which will be our future research.

## Data availability statement

Publicly available datasets were analyzed in this study. This data can be found here: https://github.com/spMohanty/PlantVillage-Dataset.

## Author contributions

WJ: Funding acquisition, Project administration, Supervision, Writing – review & editing. JL: Methodology, Visualization, Writing – original draft.

## Supplementary material

Supplementary Figure 1 Image equalization processing: **(A)** Histogram before image equalization processing **(B)** Histogram after image equalization processing.

Supplementary Figure 2 Residual block structure: **(A)** The basic residual block **(B)** ResNet50 residual block.

Supplementary Figure 3 Feature map in visual convolution.
