# Supplementary figures and images for: A ResNet50-DPA model for tomato leaf disease identification
Source: Front Plant Sci. 2023 Oct 16;14:1258658. doi: 10.3389/fpls.2023.1258658 (PMC10614023; doi:10.3389/fpls.2023.1258658)

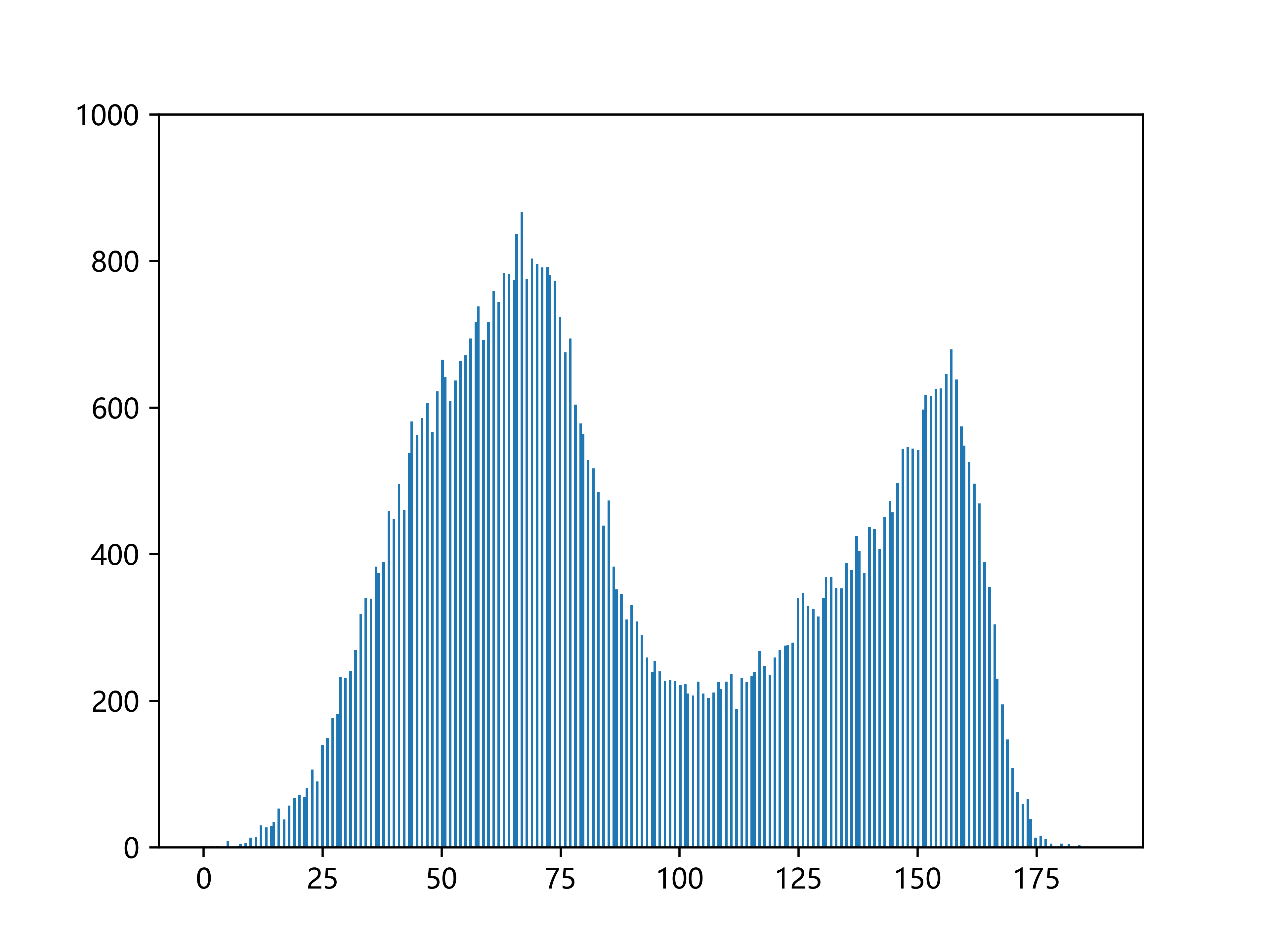

Supplement: Supplementary Figure 1 — Image equalization processing: (A) Histogram before image equalization processing (B) Histogram after image equalization processing. [file DataSheet_1.zip › Data Sheet1/Supplementary material/Figure S1a.png]

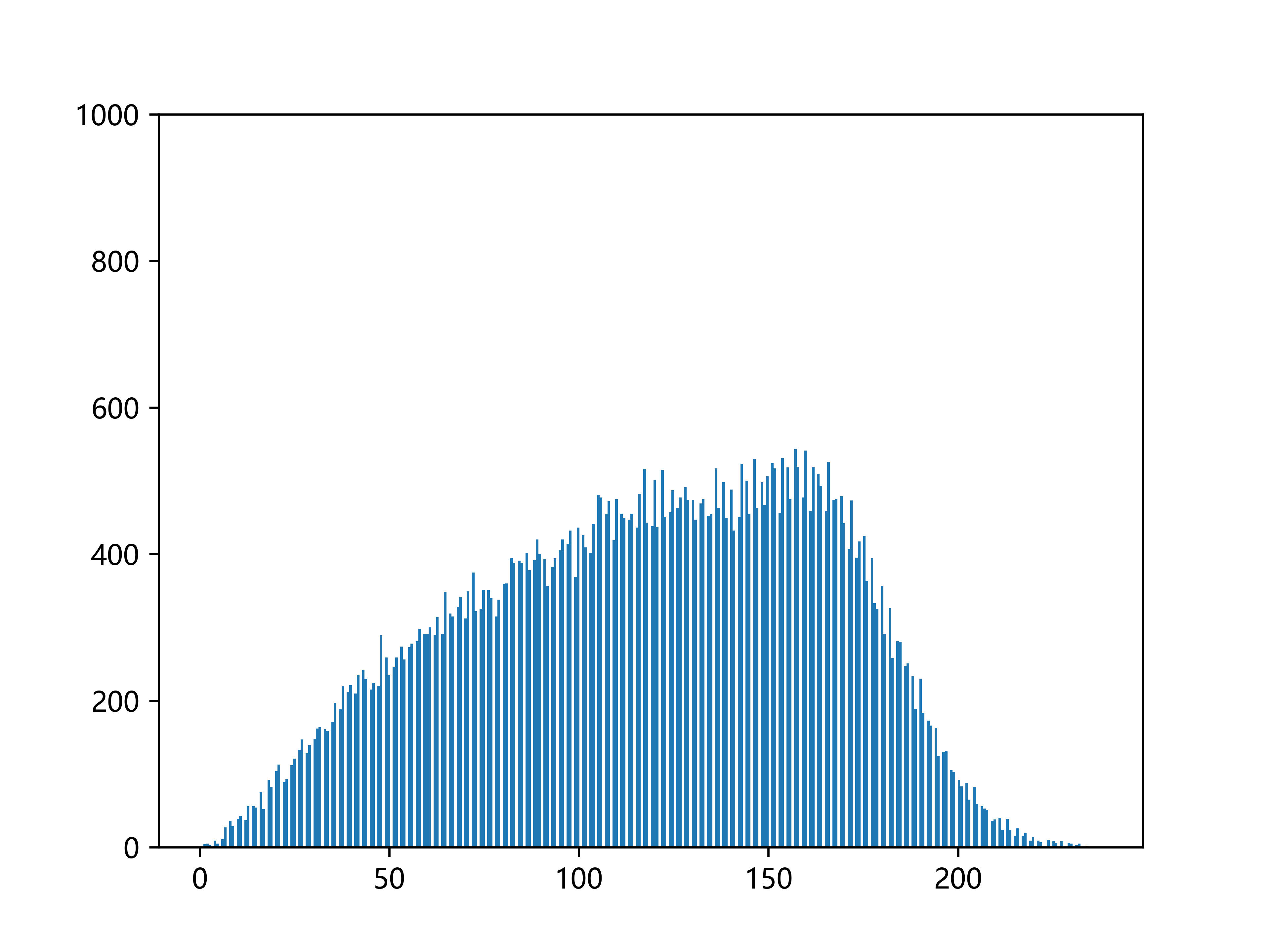

Supplement: Supplementary Figure 1 — Image equalization processing: (A) Histogram before image equalization processing (B) Histogram after image equalization processing. [file DataSheet_1.zip › Data Sheet1/Supplementary material/Figure S1b.png]

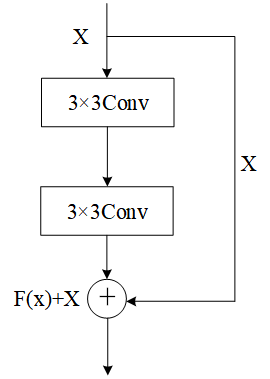

Supplement: Supplementary Figure 1 — Image equalization processing: (A) Histogram before image equalization processing (B) Histogram after image equalization processing. [file DataSheet_1.zip › Data Sheet1/Supplementary material/Figure S2a.png]

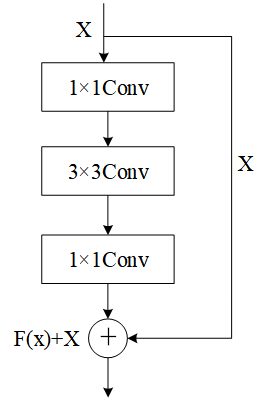

Supplement: Supplementary Figure 1 — Image equalization processing: (A) Histogram before image equalization processing (B) Histogram after image equalization processing. [file DataSheet_1.zip › Data Sheet1/Supplementary material/Figure S2b.png]

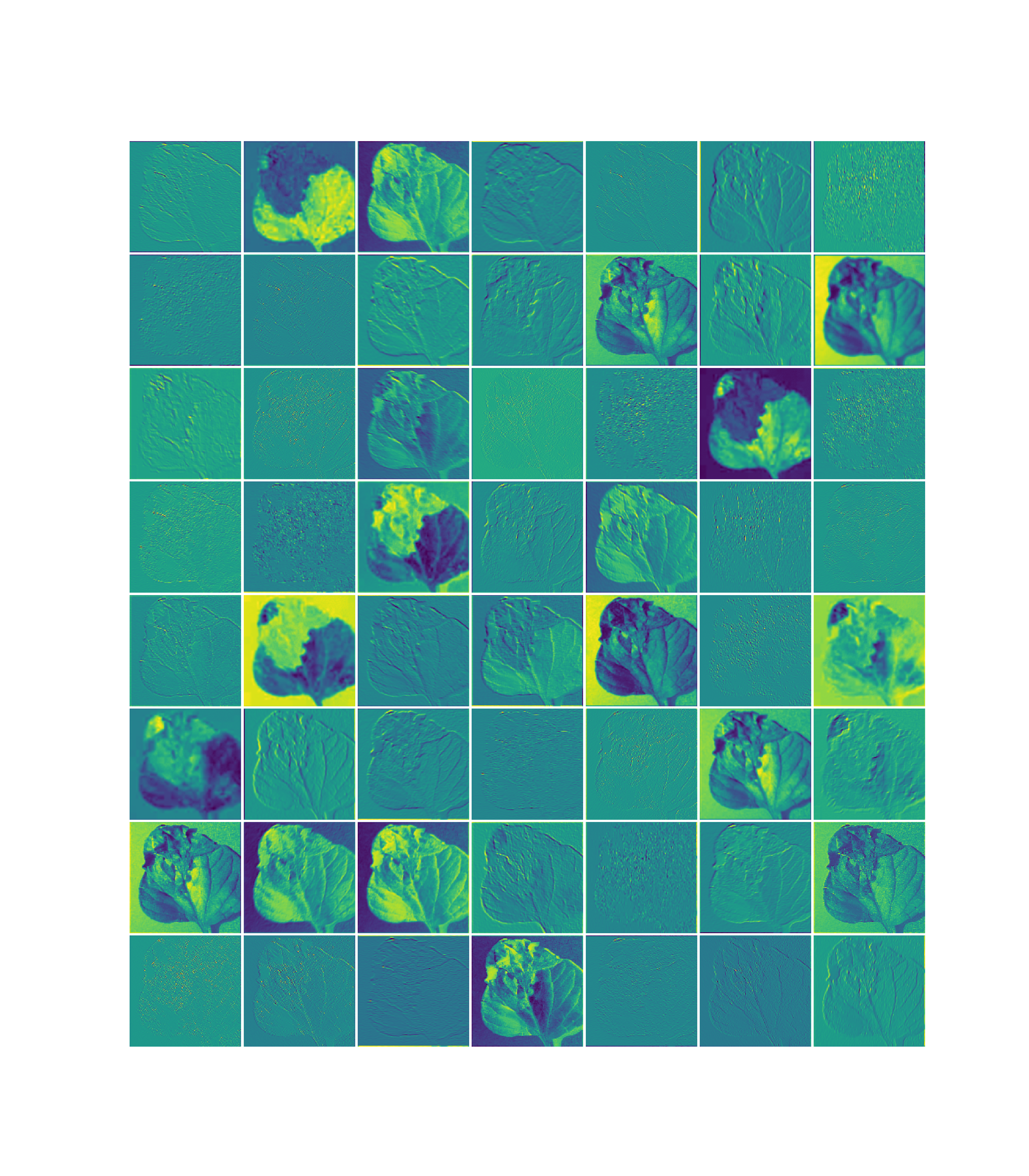

Supplement: Supplementary Figure 1 — Image equalization processing: (A) Histogram before image equalization processing (B) Histogram after image equalization processing. [file DataSheet_1.zip › Data Sheet1/Supplementary material/Figure S3a.png]

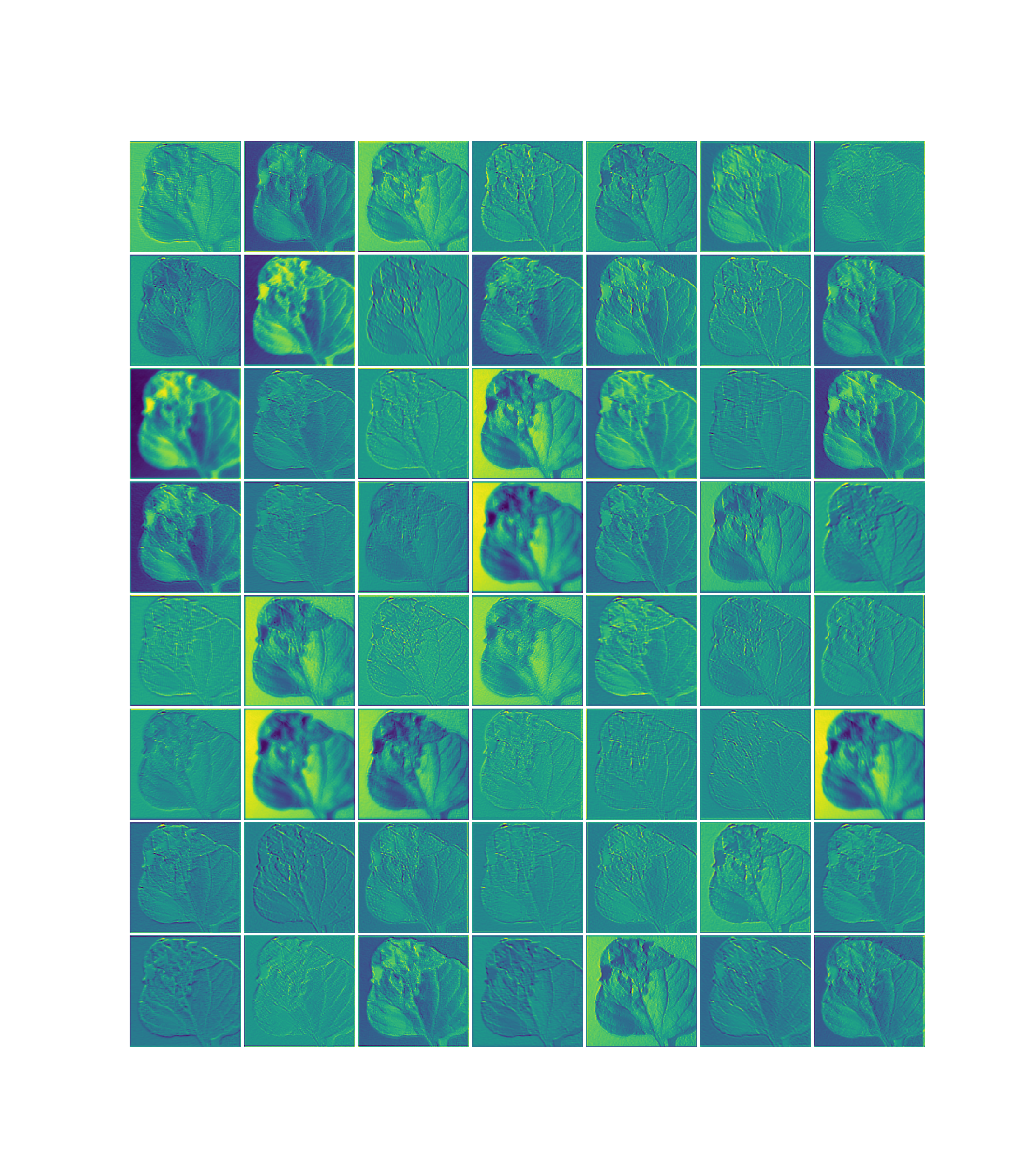

Supplement: Supplementary Figure 1 — Image equalization processing: (A) Histogram before image equalization processing (B) Histogram after image equalization processing. [file DataSheet_1.zip › Data Sheet1/Supplementary material/Figure S3b.png]

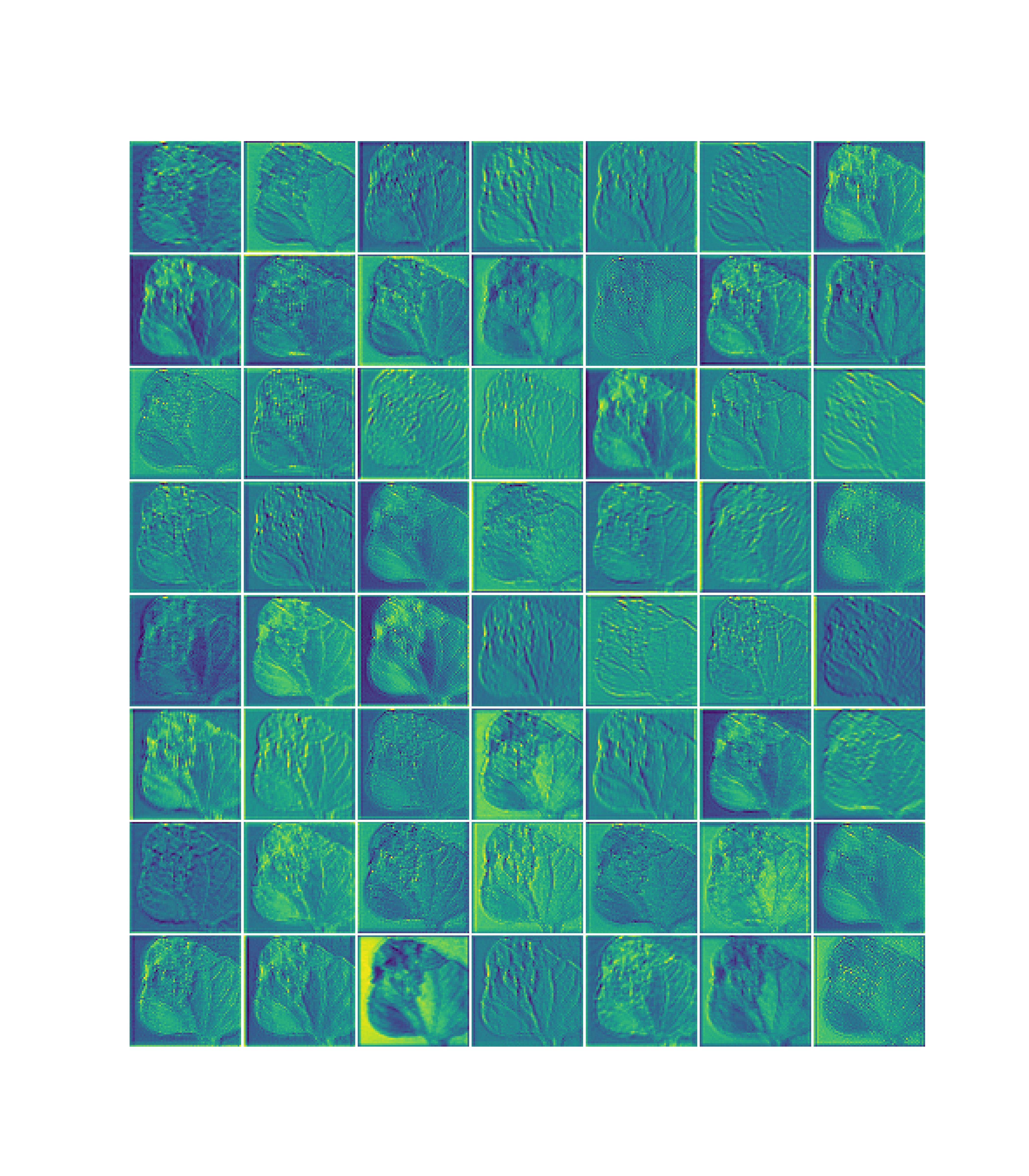

Supplement: Supplementary Figure 1 — Image equalization processing: (A) Histogram before image equalization processing (B) Histogram after image equalization processing. [file DataSheet_1.zip › Data Sheet1/Supplementary material/Figure S3c.png]

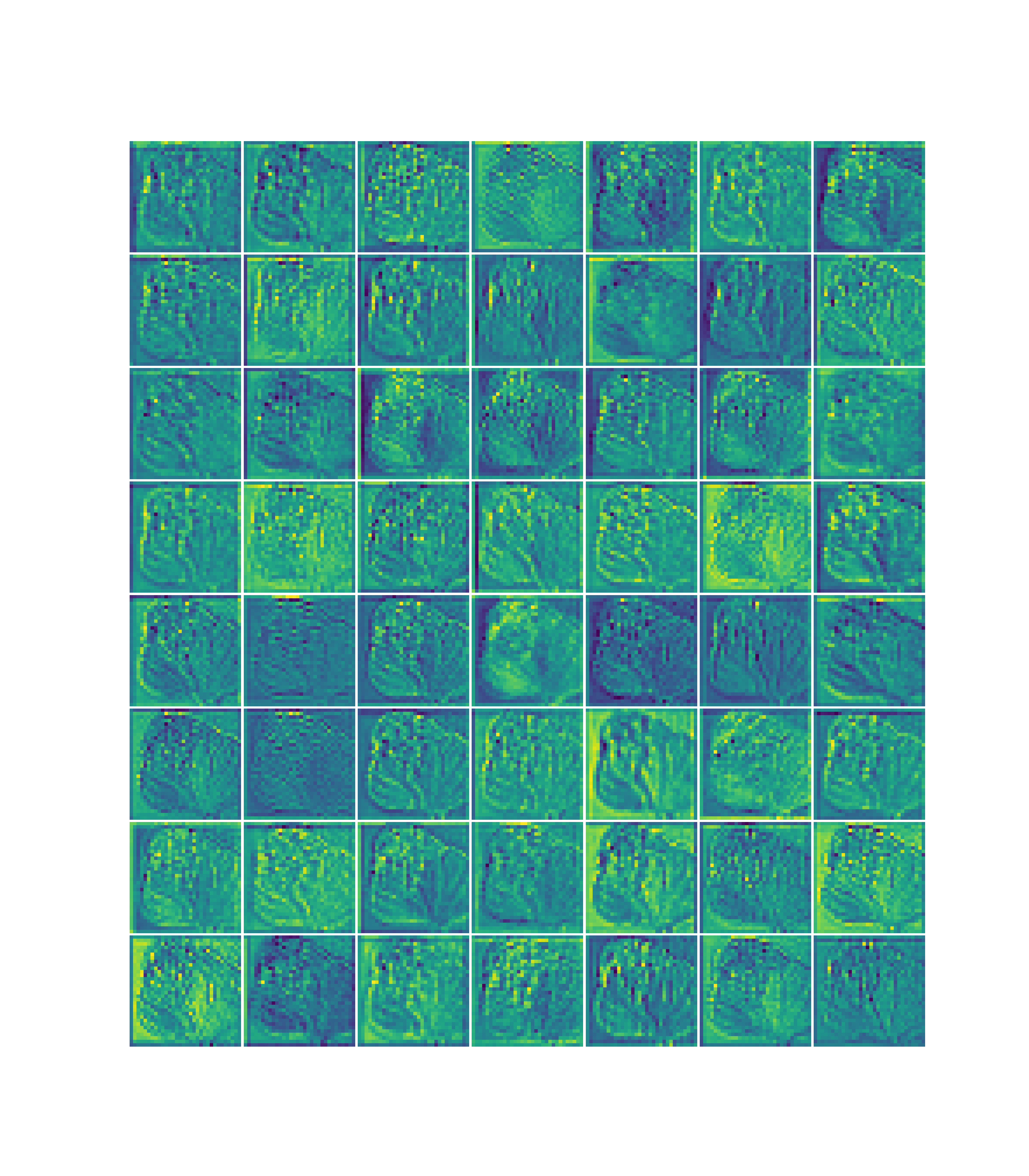

Supplement: Supplementary Figure 1 — Image equalization processing: (A) Histogram before image equalization processing (B) Histogram after image equalization processing. [file DataSheet_1.zip › Data Sheet1/Supplementary material/Figure S3d.png]

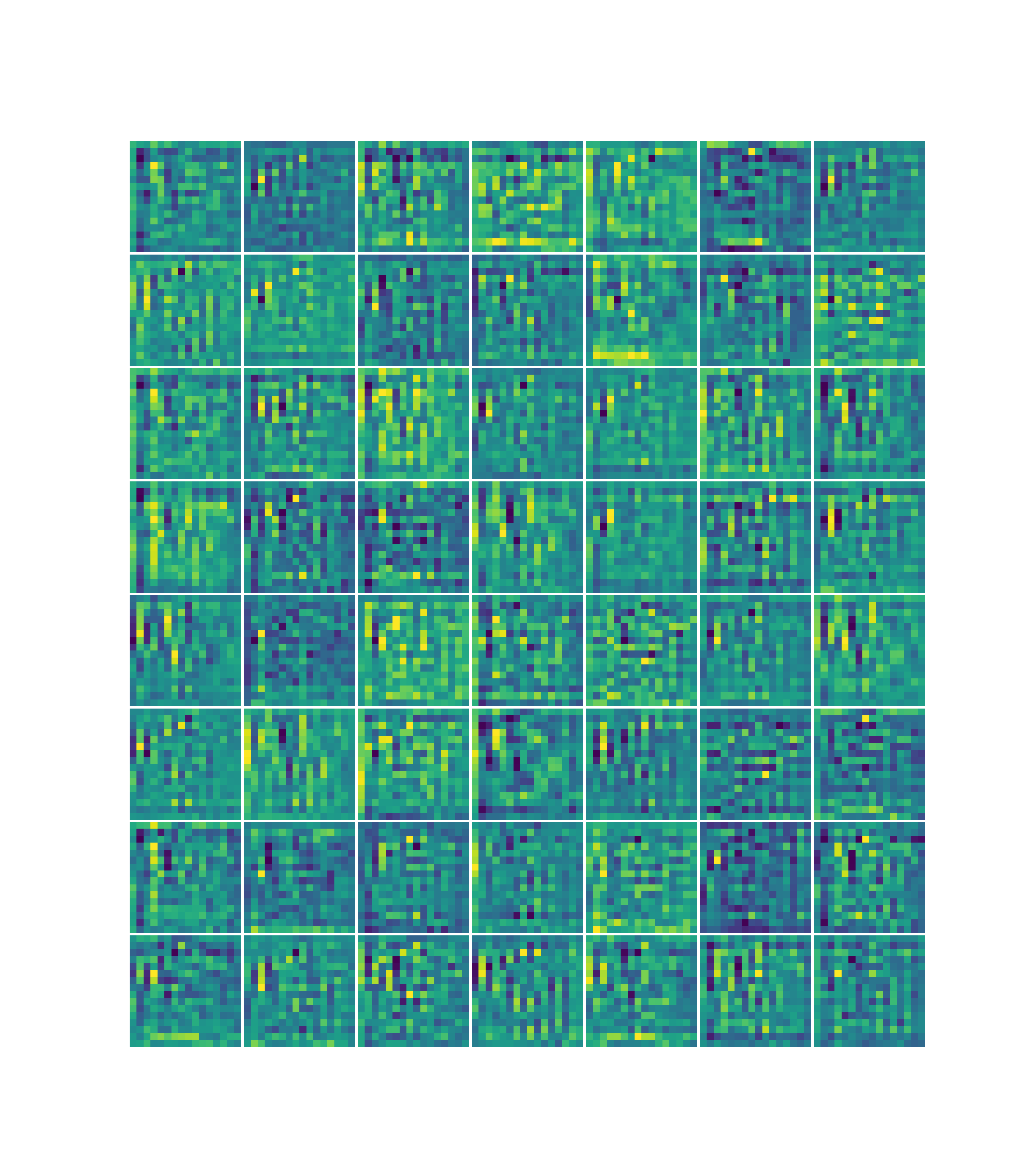

Supplement: Supplementary Figure 1 — Image equalization processing: (A) Histogram before image equalization processing (B) Histogram after image equalization processing. [file DataSheet_1.zip › Data Sheet1/Supplementary material/Figure S3e.png]

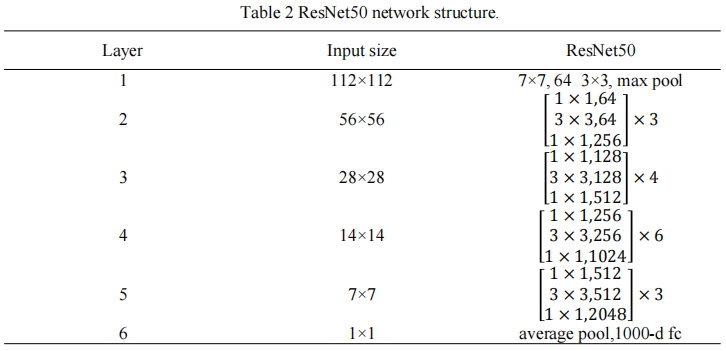

Supplement: Supplementary Figure 1 — Image equalization processing: (A) Histogram before image equalization processing (B) Histogram after image equalization processing. [file DataSheet_1.zip › Data Sheet1/Supplementary material/Table S1.png]

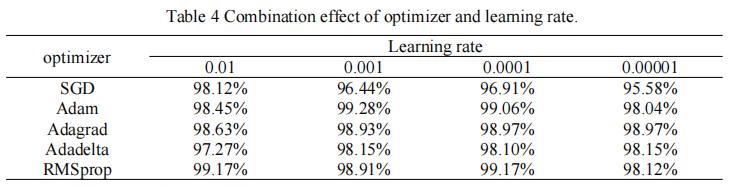

Supplement: Supplementary Figure 1 — Image equalization processing: (A) Histogram before image equalization processing (B) Histogram after image equalization processing. [file DataSheet_1.zip › Data Sheet1/Supplementary material/Table S2.png]
